# Highlight report: Tumor infiltrating lymphocytes in breast cancer

**Published:** 2019-03-04

**Authors:** Birte Hellwig

**Affiliations:** 1 Fakultät Statistik, Technische Universität Dortmund, 44221 Dortmund, Germany

## ⁯

Recently Carsten Denkert and colleagues from the Charité in Berlin published an article about the prognostic and predictive role of tumor-infiltrating lymphocytes (TIL) in neoadjuvantly treated breast cancer (Denkert et al., 2018[4]). One of the key messages of this study is that increased TIL improves response to neoadjuvant chemotherapy in all analyzed molecular subtypes (luminal-HER2-negative; HER2-positive, triple-negative). Moreover, increased TILs were associated with longer survival in HER2-positive and triple-negative breast cancer (Denkert et al., 2018[4]).

These findings confirm the results of previous studies in breast cancer. For example markers of tumor infiltrating B-cells/plasma cells have been demonstrated to predict response to neoadjuvant anthracycline-based chemotherapy in breast cancer (Schmidt et al., 2012[15]). From previous studies it has been shown that high levels of tumor infiltrating lymphocytes improve response to chemotherapy but also have a positive influence on prognosis in patients without adjuvant or neoadjuvant chemotherapy (Schmidt et al., 2012[15], 2008[14]; Heimes et al., 2017[7][8]). For example, markers of T cells and B cells are associated with longer survival in all molecular subtypes of breast cancer, such as HER2+, basal-like, luminal A, and luminal B (Heimes et al., 2017[7]). Interestingly, the prognostic effect of immune cells was most pronounced in HER2+ breast cancer (Heimes et al., 2017[7]). Several previous studies demonstrated that it is important to consider proliferation in relation to immune cell infiltration (e.g. Aaltomaa et al., 1992[1]; Schmidt et al., 2008[14], 2009[16], 2012[15]). The benefit of high immune cell infiltration was stronger in rapidly proliferating than slow proliferating tumors. Therefore, it is surprising that the study of Denkert and colleagues (2018[4]) reports that consideration of Ki67 does not change the effects of TILs. This is a discrepancy that should be revisited in future.

One of the pioneering studies on tumor infiltrating lymphocytes has been published by Aaltomaa from Kuopio in Finland (Aaltomaa et al., 1992[1]). Already in this early study the authors demonstrated that high lymphocyte infiltration in breast cancer is associated with better prognosis (Aaltomaa et al., 1992[1]). Therefore, the similarity of conclusions in previous studies (Aaltomaa et al., 1992[1]; Schmidt et al., 2018[17], 2012[15], 2008[14]) and the present manuscript demonstrate the difficulties to achieve major progress in this field of research. One of the open questions is why the consequences for prognosis of immune cell infiltrations into tumor stroma or tumor modules show such a large variability. Although generally associated with better prognosis or response to chemotherapy, TILs may also occur at high levels in patients with no response to chemotherapy or with short metastasis-free survival. Therefore, studies on TIL should routinely consider factors that influence the activity of immune cells. Besides negative immune regulators, such as PD-1, CTLA-4 (considered in breast cancer e.g. in Heimes et al., 2017[7]) numerous further factors may be influential, including the metabolic microenvironment (Marchan et al., 2017[11], 2012[12]; Lesjak et al., 2014[10]; Stewart et al., 2012[19]; Gogiashvili et al., 2018[5]; Stöber, 2017[20]; Hassan, 2017[6]), inflammatory factors and cytokines (Heimes et al., 2017[8]; Mattsson et al., 2015[13]; Sicking et al., 2014[18]), oxidative/antioxidative factors (Cadenas et al., 2010[2], 2014[3]) and ribosome related factors (Hellwig et al., 2016[9]). A further aspect in the study of Denkert et al. (2018[4]) should be considered in future studies: the authors report that luminal-HER2-negative breast cancer shows a worse prognosis with increased TILs, in contrast to all other molecular subtypes where high TILs are associated with better prognosis. However, without knowledge of details, further e.g. the differentiation of individual immune cell types, immune checkpoints and consideration of possible confounding factors this finding remains difficult to interpret. It has already been shown that depending on the tumor type the influence of e.g. T cells and B cells may differ (Heimes et al., 2017[7]). Therefore, in future studies about TILs the individual types of infiltrating cells should be considered and confounding factors, e.g. the influence of immune check-points should be included.
